# Regulation of cerebellar network development by granule cells and their molecules

**DOI:** 10.3389/fnmol.2023.1236015

**Published:** 2023-07-14

**Authors:** Muwoong Kim, Soyoung Jun, Heeyoun Park, Keiko Tanaka-Yamamoto, Yukio Yamamoto

**Affiliations:** ^1^Brain Science Institute, Korea Institute of Science and Technology (KIST), Seoul, Republic of Korea; ^2^Division of Bio-Medical Science and Technology, KIST School, University of Science and Technology (UST), Seoul, Republic of Korea

**Keywords:** cerebellum, granule cell, parallel fiber, developmental regulation, gross structure, neuronal maturation, molecule secretion, cell–cell interaction

## Abstract

The well-organized cerebellar structures and neuronal networks are likely crucial for their functions in motor coordination, motor learning, cognition, and emotion. Such cerebellar structures and neuronal networks are formed during developmental periods through orchestrated mechanisms, which include not only cell-autonomous programs but also interactions between the same or different types of neurons. Cerebellar granule cells (GCs) are the most numerous neurons in the brain and are generated through intensive cell division of GC precursors (GCPs) during postnatal developmental periods. While GCs go through their own developmental processes of proliferation, differentiation, migration, and maturation, they also play a crucial role in cerebellar development. One of the best-characterized contributions is the enlargement and foliation of the cerebellum through massive proliferation of GCPs. In addition to this contribution, studies have shown that immature GCs and GCPs regulate multiple factors in the developing cerebellum, such as the development of other types of cerebellar neurons or the establishment of afferent innervations. These studies have often found impairments of cerebellar development in animals lacking expression of certain molecules in GCs, suggesting that the regulations are mediated by molecules that are secreted from or present in GCs. Given the growing recognition of GCs as regulators of cerebellar development, this review will summarize our current understanding of cerebellar development regulated by GCs and molecules in GCs, based on accumulated studies and recent findings, and will discuss their potential further contributions.

## 1. Introduction

Neuronal network structures in the brain are precisely formed during developmental periods through intrinsic programs in individual neurons and influence from neighboring neurons. Molecular expression patterns are dynamically altered in neurons during developmental periods, as revealed by transcriptome studies (Mody et al., 2001; Saito et al., 2002; Thompson et al., 2014; La Manno et al., 2021), and these molecules likely function not only in the development of own cells but also in the development of other cells or neuronal networks. The cerebellar cortex is one of the most regularly structured brain regions and consists of three layers: the internal granular layer (IGL), the Purkinje cell (PC) layer (PCL), and the molecular layer (ML; Eccles et al., 1967). Although recent studies have discovered diversity of circuits and cell population in the cerebellum (De Zeeuw et al., 2021; Hull and Regehr, 2022), the basic structures are highly conserved throughout the cerebellum. As the sole output of the cerebellar cortex, PCs send inhibitory projections mostly to the deep cerebellar nuclei (DCN). The somas of PCs are aligned in the PCL, while their highly elaborate dendrites expand in the ML, where ML interneurons (MLIs) exist. Granule cells (GCs) are the only excitatory neurons among the major types of cerebellar neurons. Their small somas and short dendrites are located in the IGL, while their parallel fiber (PF) axons run parallel to the layer structures in the ML. In addition to the three major types of neurons, the cerebellar cortex contains several types of less abundant neurons, and their somas are located in the IGL or the PCL. The cerebellum receives two major excitatory inputs: climbing fibers (CFs) directly innervate PC dendrites in the ML, and mossy fibers (MFs) innervate GCs in the IGL. Thus, the somas, dendrites, and axons of individual types of cerebellar neurons and afferent projections from outside the cerebellum are present in the designated layers. Such organized cerebellar structures and synaptic connections are considered to be formed through well-orchestrated mechanisms during developmental periods (Park et al., 2021).

Granule cells convey information coming from MFs to PCs, making them a functionally important component in cerebellar networks. In addition to their role as components of neuronal networks, GCs have been shown to regulate the formation of cerebellar structures and networks during developmental periods (e.g., Hashimoto et al., 2009b; Legué et al., 2016; Park et al., 2019; Cadilhac et al., 2021; van der Heijden et al., 2021). The regulation is likely mediated through synaptic transmission, secreted molecules, molecular interactions, morphological constraints, or physical actions arising from the large number of GCs. Meanwhile, GCs themselves also go through dynamic events to mature (Vaudry et al., 2003; Iulianella et al., 2019; Wang and Liu, 2019; Consalez et al., 2021). In brief, GC precursors (GCPs) are originated in the rhombic lip (RL) and migrate to the external granular layer (EGL) in the cerebellum during mid-late embryonic days. GCPs then undergo intensive proliferation in the outer EGL during the first 2 weeks after birth in mice, while a portion of GCPs located in the inner EGL completes mitosis. The postmitotic GCs begin migration and simultaneously extend PFs. They first tangentially migrate at the border between the ML and the EGL, and then radially migrate along the processes of Bergmann glial cells toward the IGL. Once GCs reach the IGL, they remodel their dendrites through extension and retraction, form synapses with presynaptic MFs, and finally mature. Each GC matures through these events in turn, with all GCs reaching maturity within 3 weeks of the postnatal period in mice. Thus, GCs having PFs in the deep ML are born earlier than GCs having PFs in the superficial ML. Extensive research has been conducted on the developmental processes of GCs, leading to a comprehensive understanding of the molecules that regulate these processes, particularly in the early stages of development. These regulatory mechanisms have been thoroughly documented in various review articles (Vaudry et al., 2003; Leto et al., 2016; Lackey et al., 2018; Iulianella et al., 2019; Wang and Liu, 2019; Consalez et al., 2021). In contrast, our understanding of how GCs and their molecules regulate the structure and network formation of the cerebellum is gradually increasing. In this article, we discuss such regulation of cerebellar formation by GCs and molecules in GCs, which have been found in experimental studies using mice, unless otherwise stated. The regulation discussed here is summarized in Table 1.

**Table 1:** Summary of GC involvement in the regulation of cerebellar development.

Cerebellar developmental events		Involvement of GCs		Reference
		Mechanical or biological contributions	Molecules involved	
Size and foliation	Enlargement foliation, and lamination	Coincidental massive proliferation		Lauder et al. (1974)
		Proliferation (observation in agranular cerebellum)		Altman and Anderson (1971); Rezai and Yoon (1972); Rakic and Sidman (1973); Mariani et al. (1977); Mikoshiba et al. (1980); Herrup (1983); Herrup and Sunter (1987); Smeyne and Goldowitz (1989); Ferguson (1996); Goldowitz et al. (1997)
		Proliferation	Atoh1	Ben-Arie et al. (1997); Jensen et al. (2004); van der Heijden et al. (2021)
		Proliferation and differentiation	Wnt/β-catenin	Lorenz et al. (2011); Pei et al. (2012); Wen et al. (2013)
	Enlargement and foliation	Shh-dependent proliferation	Gli1, Gli2	Corrales et al. (2004, 2006)
		Radial migration at appropriate speed	Lkb1	Ryan et al. (2017)
		Proliferation and differentiation	CHD8	Kawamura et al. (2021); Chen et al. (2022)
	Enlargement	Proliferation	En1, En2	Orvis et al. (2012)
	Foliation	Increase in proliferation in anchoring center		Sudarov and Joyner (2007)
		Anterior–posterior orientation of cell division	CHD7	Reddy et al. (2021)
	Regulation of folia length	Differentially regulated proliferation		Legué et al. (2015); Legué et al. (2016)
		(Theoretical study) Migration at experimentally observed speed		Takeda et al. (2021)
PCs	PC migration in primordial cerebellum	Providing molecular guidance	Reelin	Mariani et al. (1977); Mikoshiba et al. (1980)
	PC monolayer formation	Accumulation of GCs in the IGL and stacking of PFs in the ML		Altman et al. (1969)
		Providing a short-range signal		Carletti et al. (2008)
			Reelin^*^	Miyata et al. (1997); Magdaleno et al. (2002)
	PF-PC synapse formation	Providing synaptic organizers that interact with GluD2 in PCs	Cbln1, NRX	Kurihara et al. (1997); Hirai et al. (2005); Matsuda et al. (2010); Uemura et al. (2010); Hashizume et al. (2013); Elegheert et al. (2016)
		PF bouton maturation for synapse formation with PCs	Mea6	Wang et al. (2021)
			Chd4	Yamada et al. (2014)
	MLI-PC synapse formation	Competition of synapse formation by forming PF synapses	Cbln1	Ito-Ishida et al. (2014)
	PC dendrite development	Supplying competing substances that activate TrkC in PCs	NT-3	Joo et al. (2014)
		Supplying competing substances that interact with GluD2 in PCs	Cbln1	Takeo et al. (2021)
		Unbiased synaptic transmission		Park et al. (2019)
	Maturation of PC firing properties	(recording in agranular mice)		van der Heijden et al. (2021)
MLIs	MLI migration	Synaptic transmission		Park et al. (2019)
		Molecular scaffolding	TAG-1	Cadilhac et al. (2021)
	Maturation	Interacting in the EGL	TAG-1	Park et al. (2019); Cadilhac et al. (2021)
		Activation of TrkB in MLIs	BDNF^*^	Rico et al. (2002)
	PF-MLI synapse formation and MLI survival	Providing synaptic organizers that interact with GluD1 in MLIs	Cbln1, NRX^*^	Yasumura et al. (2012); Konno et al. (2014)
CF inputs	Elimination of surplus CF synapses	(observation in hypogranular rats)		Crepel et al. (1981)
		PF-PC synapse formation	Cbln1	Kurihara et al. (1997); Hirai et al. (2005); Hashizume et al. (2013)
		PF-PC synaptic transmission		Chen et al. (1995); Kano et al. (1997); Offermanns et al. (1997); Kano et al. (1998)
	CF territory elongation	PF-PC synaptic transmission		Ichikawa et al. (2002); Hirai et al. (2005)
		PF-PC synaptic transmission in the deep ML		Park et al. (2019)
	CF territory segregation	PF-PC synaptic formation	Cbln1	Ichikawa et al. (2002); Hirai et al. (2005)
MF inputs	MF terminal remodeling	Coincidental GC dendritogenesis		Hámori and Somogyi (1983)
		Providing substances that promote the maturation	WNT-7a	Hall et al. (2000)
			Neuroligin	Scheiffele et al. (2000)
			FGF22	Umemori et al. (2004)
	MF-GC synapse formation	Interactions through cell adhesion molecules	Cdh7	Kuwako et al. (2014)
	Structured MF-GC synaptic connections	Coincidental development		Shuster et al. (2021); Kim et al. (2023)
Other GCs	GC radial migration	A source of glutamate^*^		Komuro and Rakic (1993)
			BDNF^*^	Wetmore et al. (1990); Rocamora et al. (1993)
	GCP proliferation in the EGL	Microenvironment of mitogenic niche^*^		Choi et al. (2005)

Abbreviations are defined in the text. Note that asterisks (^*^) in this table indicate that the molecules or mechanisms described are likely to be involved but have not been clearly identified.

## 2. Contributions of GCs and their molecules to the formation of cerebellar size and foliation

**Figure 1:**
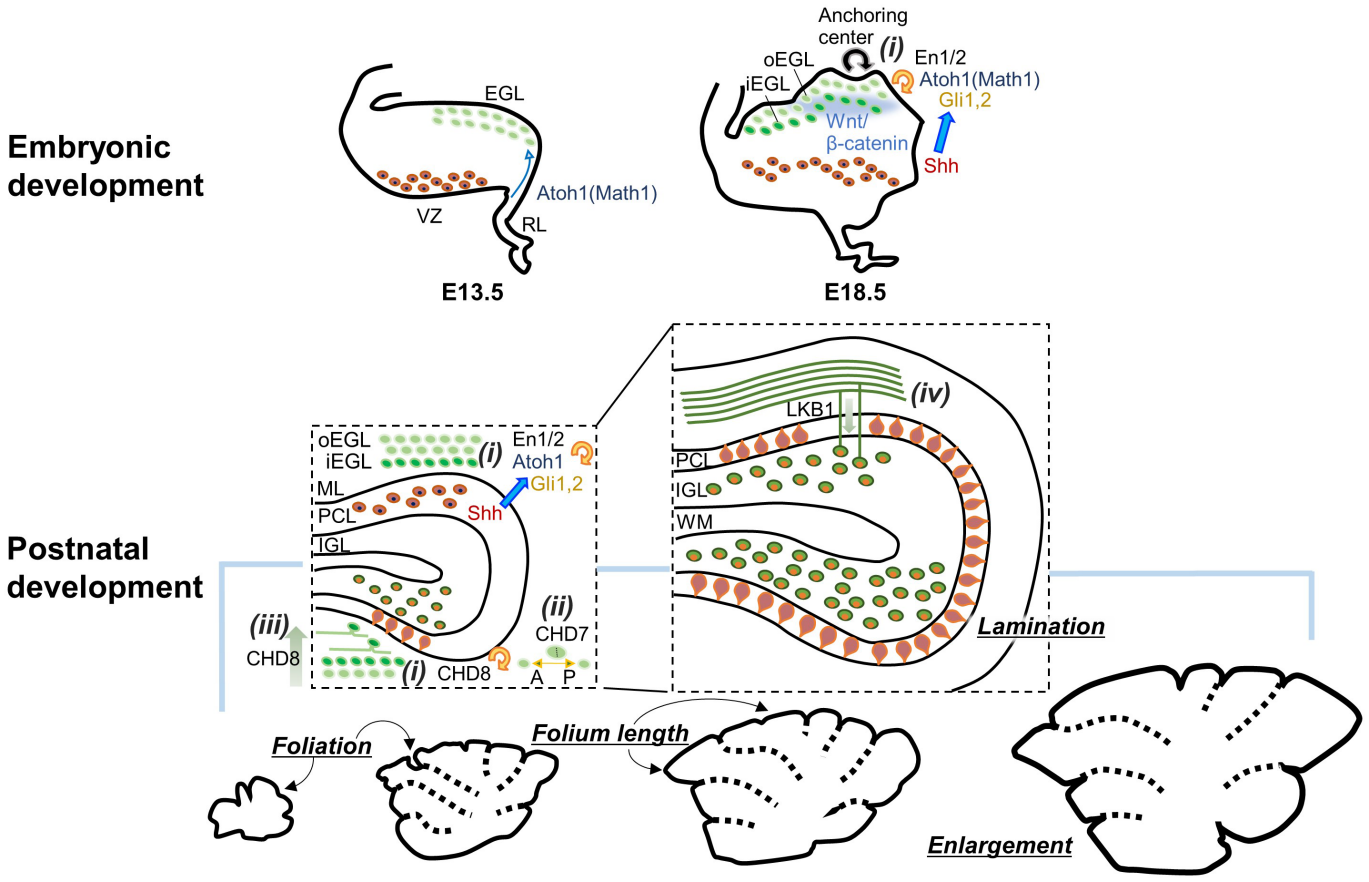
Diagram showing how GCs contribute to the formation of the cerebellar gross structures during embryonic and postnatal development. GCP proliferation **(i)**, axis of GCP division **(ii)**, GC differentiation **(iii)**, and GC migration **(iv)** affect the enlargement, foliation, and lamination of the cerebellum. Consequently, molecules involved in these GC developmental processes are also implicated. oEGL, outer EGL; iEGL, inner EGL. Other abbreviations are defined in the text.

The cerebellum, which is Latin for the “little brain,” accounts for more than 10% of the total brain volume in mice (Angenstein et al., 2007; Ma et al., 2008). The cerebellum has a complex 3D structure, with lobules divided by fissures along the anterior–posterior (a-p) axis. The lobules in the cerebellar vermis of rodents are grouped into four zones: the anterior zone (lobules I–V), the central zone (lobules VI–VII), the posterior zone (lobule VIII and anterior lobule IX), and the nodular zone (posterior lobule IX and lobule X), based on specific gene expression (Ozol et al., 1999). Despite varying sizes and shapes in individual lobules, the tri-layered cytoarchitecture with specific cell types present in designated layers is consistent throughout the cerebellum. These cerebellar gross structures begin to form in the late embryonic stage and continue to develop during the first 2 weeks after birth. This coincides with the massive proliferation of GCPs in the EGL (Lauder et al., 1974), suggesting a link between the two events. Indeed, numerous studies have demonstrated that the proliferation of GCPs is crucial not just for cerebellar enlargement but also for the formation of gross structures (Figure 1). Early studies used animals with severely reduced numbers of GCs (hypogranular or agranular cerebellum), including scrambler, weaver, reeler, and staggerer spontaneous mutation mice or x-irradiated rats, and found abnormal foliation and lamination in the cerebellum of these animals (Altman and Anderson, 1971; Rezai and Yoon, 1972; Rakic and Sidman, 1973; Mariani et al., 1977; Mikoshiba et al., 1980; Herrup, 1983; Herrup and Sunter, 1987; Smeyne and Goldowitz, 1989; Ferguson, 1996; Goldowitz et al., 1997). Based on the observation of both macroscopic and microscopic morphogenesis of the cerebellum, it was further demonstrated how GCP proliferation contributes to foliation. Foliation was initiated at the late embryonic day 17.5 (E17.5) by the increase in GCP proliferation and consequent inward thickening of the EGL in the anchoring centers, which are regions that will become the base of fissures in the future (Sudarov and Joyner, 2007). At later developmental stages, differentially regulated GCP proliferation in different lobules appears to be involved in determining the different shapes and lengths of cerebellar lobules (Legué et al., 2015, 2016). Thus, GCP proliferation and their locally differential control largely contribute to the formation of unique cerebellar gross structures, although the determinants of locally differential control remain uncertain.

Similar to other neurons (Hevner, 2006; Hevner et al., 2006; Stevanovic et al., 2021), early GC development is regulated by several types of transcription factors and their regulating molecules (see reviews in Wang and Liu, 2019; Consalez et al., 2021). Because cerebellar lobule and folium formation depend on the expansion of the GC population, these molecules in GCs have been shown to be essential not only for GC proliferation, survival, or neurogenesis, but also for the formation of the gross structure of the cerebellum (Figure 1i). One of the molecules for which such a link has been described is the atonal basic helix–loop–helix transcription factor 1 (Atoh1, Math1), which is expressed in the RL from E13. In mice lacking Atoh1, GCs were not produced, and the EGL was not formed, leading to severely altered cerebellar gross structures without foliation and lamination (Ben-Arie et al., 1997; Jensen et al., 2004; van der Heijden et al., 2021). Sonic hedgehog (Shh) produced in PCs has long been known as a potent inducer of GCP proliferation and as an important factor for appropriate folium formation in the cerebellum (Dahmane and Altaba, 1999; Lewis et al., 2004). Three Gli proteins (Gli1, Gli2, and Gli3), zinc-finger transcription factors participating in the Shh signaling pathway (Carballo et al., 2018), are expressed in the EGL (Corrales et al., 2004). Considering the reduced foliation in mice lacking Gli2 and the further reduction of foliation in mice lacking both Gli1 and Gli2 (Corrales et al., 2004, 2006), Gli1 and Gli2 are likely to cooperate as targets of Shh in GCPs to promote proliferation and consequent folium formation. Apart from its role in GCP production during embryonic days, Atoh1 promotes GCP proliferation during postnatal development by controlling cilia formation required for Shh signaling (Flora et al., 2009; Chang et al., 2019). Thus, Atoh1 and the Shh signaling pathways likely cooperate in cerebellar folium formation during postnatal development. In addition to the Shh signaling pathway, the Wnt/β-catenin signaling pathway has been shown to be involved in folium and layer formations through regulating GC development (Lorenz et al., 2011; Pei et al., 2012; Wen et al., 2013). Both the absence and enhancement of Wnt/β-catenin signaling in GCPs led to abnormal cerebellar gross structures and reduced cerebellar size, although they caused different changes at the cellular level: the absence of signaling facilitated GCP proliferation, leading to an accumulation of abnormally matured GCs near the pial surface, whereas increasing signaling inhibited GCP proliferation, leading to a reduction of GCs. Homeobox transcription factors, Engrailed 1 (En1) and 2 (En2), have also been implicated in cerebellar folium formation (Bilovocky et al., 2003; Sudarov and Joyner, 2007; Cheng et al., 2010). However, based on a study that used En1/2 conditional knockout mice either in the RL or the ventricular zone (VZ), the latter of which gives rise to cerebellar γ-aminobutyric acid (GABA)-ergic neurons, their expression in GCs appears to contribute more to cerebellar enlargement than to the regulation of foliation (Orvis et al., 2012).

The abovementioned example of the absence of Wnt/β-catenin signaling raises a possibility that the formation of cerebellar gross structures relies not only on the expansion of the GC population but also on the proper maturation of GCs. In line with this concept, multiple studies have demonstrated that cerebellar foliation is influenced by events in GC development beyond just proliferation (Figures 1ii–iv). The absence of liver kinase B1 (Lkb1), also known as serine/threonine kinase 11, in GCPs was shown to increase cerebellar size and foliation without affecting proliferation, but through delayed radial migration of GCs (Ryan et al., 2017). A theoretical study also predicted that GC migration at the experimentally observed speed (Yacubova and Komuro, 2002) resulted in non-uniform GC accumulation in the IGL and consequent folia lengthening (Takeda et al., 2021). Moreover, cerebellar gross structures are also regulated by two chromodomain helicase DNA-binding (CHD) proteins, CHD7 and CHD8, which are associated with cerebellum-related neurodevelopmental disorders. CHD7 is a major causative molecule of CHARGE syndrome, and CHD8 is a risk factor for autism spectrum disorder. Deletion of CHD8 in GCPs induced defects in foliation and hypoplasia through the attenuated proliferation and precocious differentiation of GCs (Kawamura et al., 2021; Chen et al., 2022). The conditional knockout of CHD7 in GCPs resulted in a unique cerebellar structure with polymicrogyria and reduced anterior–posterior foliation (Reddy et al., 2021). Interestingly, the CHD7 deletion did not alter GCP proliferation, GC migration, and neurite development, but altered the preferred axis of division of GCPs from anterior–posterior orientation to mediolateral orientation, supporting the idea that the axis of GCP division is important for the formation of cerebellar foliation (Legué et al., 2015; Lejeune et al., 2019).

## 3. Contributions of GCs and their molecules to cerebellar development at the cellular and synaptic levels

When the size and gross structures of the cerebellum are altered during postnatal development, the neuronal morphology also undergo changes through proliferation, differentiation, migration, dendritogenesis, and synaptogenesis. Consequently, the formation of the cerebellar network is typically completed by around 3–4 weeks of age. While GCs are a major factor of the macroscopic alterations in the developing cerebellum, as described above, they also serve as regulators of cerebellar network formation through their mechanical, morphological, functional, or molecular influences on neuronal development and synaptic formation. In this section, we aim to introduce the implications of GCs for the development of individual components and to further predict possible functions or relevance of GCs for their development based on recent studies. For clarification, we simply describe the developmental processes of individual components at the beginning of each subsection, and then describe the contributions of GCs to these processes.

### 3.1. PC development

Purkinje cells are generated from the VZ of the cerebellar anlage and undergo their final mitosis around E10–E13 in mice (Miale and Sidman, 1961; Hatten and Heintz, 1995; Sotelo and Rossi, 2013). The newborn PCs migrate toward the pial surface of the primordial cerebellum and form a multilayered structure called the PC plate (Hatten and Heintz, 1995; Miyata et al., 2010; Sotelo and Rossi, 2013). PC somas are then organized into a monolayer by the end of the first postnatal week (Sotelo and Rossi, 2013). While PC axonal projections to the DCN appear to be formed during embryonic periods, PC dendritic morphology is dynamically remodeled during postnatal development through growth, branching, and regression (Armengol and Sotelo, 1991; Kapfhammer, 2004; Sotelo and Rossi, 2013; Beekhof et al., 2021). Such dendritogenesis leads to the characteristic structure of highly arborized PC dendrites mostly with one or two primary dendrites (Cerminara et al., 2015). During the second and third postnatal weeks when dendritogenesis actively occurs, PCs also establish synaptic connections with two excitatory inputs, PFs and CFs, and inhibitory inputs from MLIs (Sassoè-Pognetto and Patrizi, 2017).

**Figure 2:**
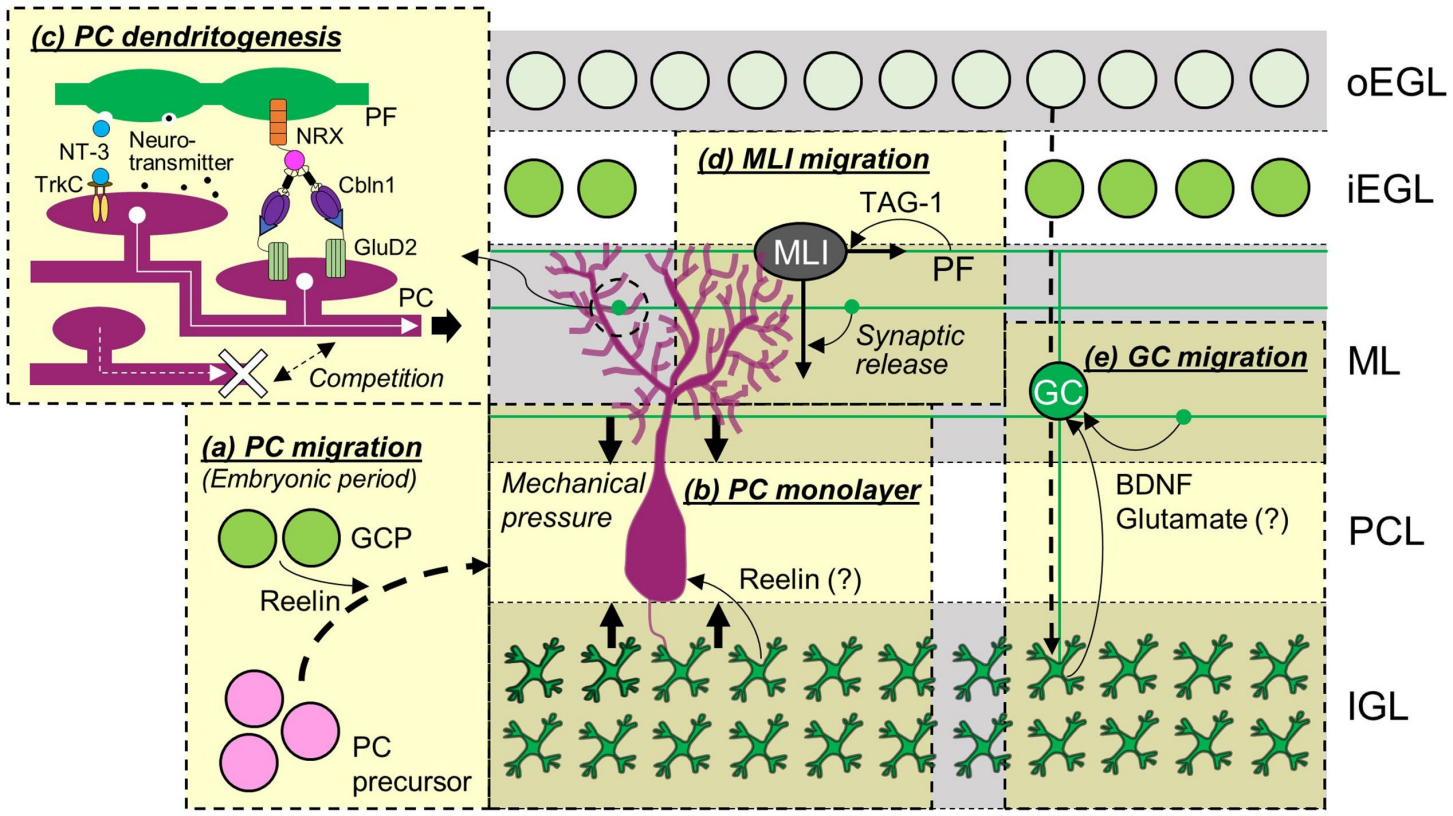
Contributions of GCs to the development of cerebellar neurons. The migration of PC precursors during embryonic periods **(A)**, formation of PC monolayers **(B)**, development of PC dendrites **(C)**, and migration of MLI **(D)** and other GCs **(E)** are regulated by GCs and molecules released from or expressed in GCs. Abbreviations are defined in the text.

Granule cells can influence PC development from the moment PCs migrate in the primordial cerebellum (Figure 2A). The PC migration toward the pial surface is mediated by an extracellular glycoprotein, Reelin, and as a consequence, PCs remained in the central area of the cerebellum in mice lacking *reelin* (Mariani et al., 1977; Mikoshiba et al., 1980). There are two sources of Reelin around E13 (Miyata et al., 1996; Schiffmann et al., 1997; Rice and Curran, 2001), the EGL and the nuclear transitory zone (NTZ), the latter of which presumably includes future DCN neurons (Fink et al., 2006; Elsen et al., 2013). In Atoh1-null mice that lack the EGL, a significant subpopulation of PCs failed to migrate from the central area to the pial surface (Ben-Arie et al., 1997; Jensen et al., 2002). In addition, abnormal PC positions in cultured slices obtained from mice lacking Reelin were restored by co-culturing with Reelin-positive GCs (Miyata et al., 1997). These data indicate that normal PC migration requires Reelin originating not only from the NTZ but also from GCPs in the EGL. The formation of the PC monolayer during the first postnatal week also relies on GCs (Figure 2B). A concept was proposed that monolayer formation is mediated by the mechanical pressure due to the accumulation of GCs in the IGL and simultaneous stacking of their PFs in the ML (Altman and Winfree, 1977), based on the observation of PC soma misalignment in hypogranular cerebella caused by X-ray irradiation (Altman et al., 1969). The positions of PCs transplanted into the developing cerebellum were affected by the locations of the EGL at the time of PC transplantation (Carletti et al., 2008), indicating that interactions with the EGL via short-range signals determine the positions of PC somas. The signals may include Reelin, because two studies suggested that Reelin secreted from GCs may play roles in the formation of the PC monolayer (Miyata et al., 1997; Magdaleno et al., 2002). This idea is reasonable, considering that Reelin is present in the EGL from the embryonic period to the first postnatal week (Miyata et al., 1996).

**Figure 3:**
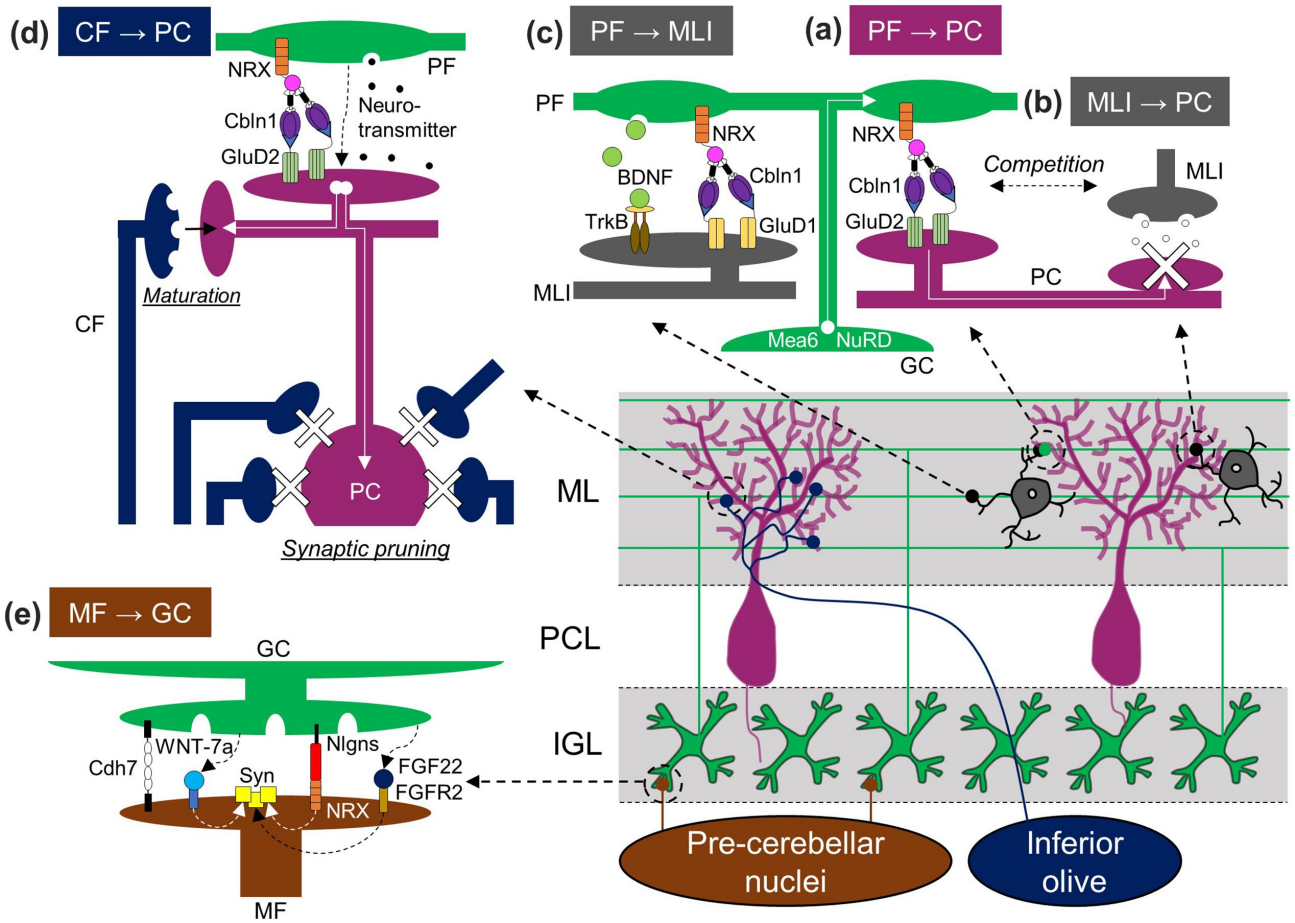
GC-dependent regulation of synaptic formation in the cerebellum. GCs influence the establishment not only of GC synapses [PF-PC **(A)**, PF-MLI **(C)**, and MF-GC **(E)** synapses], but also other synapses [MLI-PC **(B)** and CF-PC **(D)** synapses] through the release of molecules, presentation of membrane molecules, synaptic activation, or physical competition. Nlgns, Neuroligin. Other abbreviations are defined in the text.

Among the events that take place later in PC development, synaptic formation between PFs and PCs largely relies on molecules released from PFs or expressed in GCs (Figure 3A). A C1q family protein, Cbln1, is released from PFs through a unique, activity-dependent mechanism, which is a release from lysosomes (Ibata et al., 2019). Cbln1 then forms a tripartite complex with neurexin (NRX) expressed in PFs and glutamate receptor δ2 (GluD2) expressed in PC dendrites, leading to the formation and maintenance of PF-PC synapses (Matsuda et al., 2010; Uemura et al., 2010; Elegheert et al., 2016). This NRX/Cbln1/GluD2 tripartite complex appears to regulate the clustering of postsynaptic molecules, such as AMPA-type glutamate receptors or Homer 3, in PCs (Matsuda et al., 2010). GC-specific deletion of meningioma expressed antigen 6 (Mea6), initially found in tumor cells (Heckel et al., 1997), resulted in a reduction of PF-PC synapse formation, presumably due to impaired intracellular transportation of molecules required for synapse formation, including vesicular glutamate transporter 1 (vGluT1) and brain-derived neurotrophic factor (BDNF; Wang et al., 2021). PF-PC synapse density was also found to be reduced in mice lacking Chd4, a subunit of the nucleosome remodeling and deacetylation (NuRD) complex, specifically in GCs (Yamada et al., 2014). These observations suggest the functions of Mea6, Chd4, the NuRD complex, or their downstream molecules in GCs for PF-PC synaptogenesis. In addition, the maturation of synapses between CFs and PCs is highly dependent on PFs, which will be elaborated in the subsequent section regarding major afferent pathways (section 3.3). Conversely, while GCs appear to be involved in the maturation of MLIs, as described below (section 3.2), and this, in a broader context, could be interpreted as their contribution to the formation of inhibitory synapses from MLIs to PCs, their specific role in the process of inhibitory synapse formation itself remains uncertain. A study demonstrated that the absence of Cbln1 led to an increase in MLI-PC synapses, and this increase was reversed by the addition of recombinant Cbln1 in a GluD2-dependent manner (Ito-Ishida et al., 2014). Because Cbln1 and GluD2 are critical for PF-PC synapse formation, this study raises the possibility that PF synapses may engage in competitive interactions with MLI inhibitory synapses for PC dendrites through the release of Cbln1, which binds to GluD2 (Figure 3B). However, a partial blockade of transmitter release from PFs located in the middle ML did not affect the distribution of MLI synapses (Park et al., 2019), indicating that the competitive interactions would not be mediated by PF synaptic transmission.

Even though studies in cultured cerebellar neurons demonstrated the role of GCs in PC dendrite outgrowth (Baptista et al., 1994; Morrison and Mason, 1998; Hirai and Launey, 2000), GCs were originally considered to be inessential for PC dendrite development *in vivo*. This is because PC dendrite morphology was not altered when PF-PC synapses were globally impaired in transgenic mice expressing tetanus toxin (TeTx) in GCs (Kim et al., 2009) or in knockout mice lacking GluD2 (Kashiwabuchi et al., 1995; Kurihara et al., 1997), type 1 metabotropic glutamate receptor (mGluR1; Kano et al., 1997), or Cbln1 (Hirai et al., 2005). In contrast, clearly abnormal PC dendrites have been observed with partial impairment of GC-PC interaction (Figure 2C). Sparse knockout of the neurotrophin receptor tropomyosin-related kinase C (TrkC) in PCs, but not global knockout of TrkC, reduced the complexity of PC dendritic arborization (Joo et al., 2014). The phenotype was rescued by additional removal of its ligand, neurotrophin-3 (NT-3), from GCs. Similarly, sparse but not global knockout of GluD2 resulted in abnormal PC dendrite morphology, with under-elaboration in the deep ML and overelaboration in the superficial ML, which was rescued by additional removal of the GluD2 interacting partner, Cbln1, secreted from GCs (Takeo et al., 2021). In simple terms, these studies demonstrated that a sparse reduction of TrkC or GluD2 in PCs resulted in abnormal PC dendrite morphology due to a lack of interaction with NT-3 or Cbln2 secreted by GCs, indicating that molecules secreted by GCs actually play a crucial role in PC dendrite morphogenesis. In particular, these specific molecules are not universally essential but rather exert their influence through competition-based mechanisms. In this process, neighboring PCs appear to compete for binding to these molecules, which ultimately affects the dendritic arborization of each PC. Supporting this idea that molecules secreted by GCs contribute to PC dendrite morphogenesis, blocking transmitter release by expressing TeTx specifically in PFs located in the middle ML led to the local reduction of PC dendritic branches (Park et al., 2019). Thus, GCs likely regulate PC dendrite development by providing molecules that promote normal dendritic branching and outgrowth.

Although Atoh1-null mice have often been used to test the importance of GCs in cerebellar embryonic development, they are neonatal lethal and cannot be used to test postnatal functional development. On the other hand, conditional knockout mice lacking Atoh1 in En1-expressing cells are useful, because they remain viable during the second postnatal week and still have an agranular cerebellum. While PC firing patterns were dynamically altered during postnatal developmental periods in control mice (Beekhof et al., 2021; van der Heijden et al., 2021), conditional Atoh1 knockout mice maintained an immature state of firing patterns (van der Heijden et al., 2021). Thus, GCs are also critical for the maturation of PC firing properties. Overall GC-mediated regulation of structural and functional PC development during postnatal periods appears to be achieved through several mechanisms, including synaptic organizers, neurotrophin release, or synaptic transmission, as described above. It was previously shown that the presynaptic coupling between calcium channels and the sensor for vesicle fusion at PF boutons changes from a loose microdomain to a tight nanodomain around the second or third postnatal week (Baur et al., 2015; Kusch et al., 2018; Schmidt, 2019). Considering the temporal coincidence, it is possible to speculate that synaptic transmission via the loose microdomain at PF boutons is appropriate for the regulation of PC development.

### 3.2. MLI development

Molecular layer interneurons, consisting of two types of neurons, basket and stellate cells, provide a feedforward inhibition motif by receiving inputs from GCs and sending inhibitory signals to PCs. MLIs are part of the cerebellar GABAergic interneurons derived from specific progenitor populations in the VZ that differ from PC progenitor populations (Leto et al., 2012; Prestori et al., 2019). Progenitors of GABAergic interneurons continue to proliferate in the prospective white matter (pWM) from late embryonic days to the second postnatal week. According to the inside-out sequence of differentiation, MLIs are finally born in the first to second postnatal week (Leto et al., 2016). Immature postmitotic MLIs then travel toward their final destinations by taking a complex migratory route via radial and tangential migration in the ML (Simat et al., 2007; Cameron et al., 2009).

To the best of our knowledge, no studies have been reported regarding the question of whether GCs or molecules released from GCs are involved in the developmental process of MLIs in the VZ or pWM. It has been shown that GABAergic interneurons, including MLIs, differentiate into mature identities under the influence of local environmental cues existing in the pWM (Leto et al., 2006, 2009, 2012). Although the nature or source of such cues has not been identified, one possible involvement of GCs in MLI development in the pWM might be the provision of the cues, since many GCs already exist in the IGL, right next to the pWM, at the time when MLIs acquire their identities. In contrast to the early developmental process of MLIs, the complex migration of MLIs is at least partially regulated by GCs (Figure 2D). This was first suggested by a study showing that glutamatergic synaptic transmission was involved in MLI migration (Wefers et al., 2017). A specific blockade of synaptic transmission from PFs resulted in abnormal distributions of MLIs (Park et al., 2019), providing direct evidence of the involvement of GC-dependent synaptic transmission in the positioning of MLIs, likely through regulating their migrations. Furthermore, a recent study demonstrated an interesting interaction between immature GCs and migrating MLIs (Cadilhac et al., 2021). Once late-born MLIs migrate to the EGL, they stop radial migration and start tangential migration at the inner EGL. The study demonstrated that this tangential migration was supported by PFs of premigratory GCs and TAG-1 expressed in such immature PFs.

In addition to MLI migration, GCs and molecules in GCs appear to promote the functional and structural maturation of MLIs. After deleting Atoh1 from GCPs in the first postnatal week, which resulted in the depletion of GCPs in the EGL, the expression of parvalbumin, a mature MLI marker, was absent in MLIs located in the outer ML, and the levels of glutamic acid decarboxylase (GAD) 65, a GABAergic presynaptic marker, were reduced in the ML (Cadilhac et al., 2021). The reduction of GAD65 in the ML was also observed when a neurotrophin receptor, TrkB, was depleted in the cerebellum (Rico et al., 2002). Although the source of neurotrophins regulating MLI synaptic differentiation is not completely clarified, BDNF released from GCs is likely involved in the regulation. BDNF is one of the two major neurotrophins activating TrkB (Huang and Reichardt, 2001), and its mRNA is specifically expressed in GCs within the rat cerebellar cortex during postnatal developmental periods (Rocamora et al., 1993), although immunohistochemical BDNF signals were detected in PCs of adult mice (Cook et al., 2022). Similar to PF-PC synapses, PF-MLI synapses are believed to be formed via a tripartite trans-synaptic bridge (Andrews and Dravid, 2021), because MLIs express GluD1, which can also form a tripartite trans-synaptic bridge with Cbln1 and NRX (Yasumura et al., 2012), and GluD1 knockout mice showed a reduction of PF-MLI synapses (Figure 3C, Konno et al., 2014). In the GluD1 knockout mice, the size and number of MLIs were reduced (Konno et al., 2014). Thus, GCs appear to play a role in the regulation of neuronal maturation and survival of MLIs through physical interaction in the EGL, neurotrophin release, or synaptic formation with MLIs.

### 3.3. Major afferent pathways

Climbing fibers originating from the inferior olivary nucleus arrive in the developing cerebellum around E14–E15 (Reeber et al., 2013; Rahimi-Balaei et al., 2015) and make immature synapses onto PCs by P3 (Hashimoto and Kano, 2013; Kano et al., 2018). During the early postnatal stages known as the creeper stage and pericellular nest stage (Chedotal and Sotelo, 1993), multiple CFs form contacts with immature PC dendrites and PC somas (Hashimoto et al., 2009a). The CF-PC synapses are then dynamically rearranged to create characteristic mono-innervation. To generate such mature CF-PC synapses, one CF is strengthened and forms a few hundred synapses on PC dendrites, while surplus CFs are gradually eliminated through two distinct steps, early and late elimination.

In the agranular cerebellar model, vGluT2 staining signals were present around PC somas, and complex spikes were detected from PCs, both of which are signs of the existence of CF synapses (van der Heijden et al., 2021). This suggests that GCs are not required for early CF development, namely, the arrival to the cerebellum and the formation of immature synapses. In contrast, several stages of CF synapse maturation rely on GCs (Figure 3D). GCs are well known to be required for the elimination of surplus CFs. Multiple CF innervations remained in the mature cerebellum of hypogranular rats (Crepel et al., 1981; Bailly et al., 2018) or knockout mice lacking molecules required for the functional or structural formation of PF-PC synapses, such as mGluR1, protein kinase Cγ, phospholipase Cβ4, α-subunit of heterotrimeric Gq protein, or GluD2 in PCs, and Cbln1 in GCs (Chen et al., 1995; Kano et al., 1997, 1998; Kurihara et al., 1997; Offermanns et al., 1997; Hirai et al., 2005; Hashizume et al., 2013). In addition to the elimination of surplus CFs, GCs also contribute to the strengthening and maturation of the remaining CFs, known as winner CFs. The strengthening and maturation involve complex processes, such as the elongation of CF synapse territories along PC dendrites, the reorganization of CF synapses within the CF territories, and the segregation of CF territories from PF territories (Ichikawa et al., 2016). Consequently, the involvement of GCs appears to be complex. In mice lacking mGluR1, the CF territory was reduced, and CF and PF territories remained largely mixed (Ichikawa et al., 2016). Considering that mGluR1 mainly functions at PF synapses due to the glutamate transporters limiting mGluR1 responses at CF synapses (Dzubay and Otis, 2002), the results suggest the requirement of PF inputs for CF territory elongation and territory segregation. Despite reduced CF territories when PF inputs were functionally impaired, CF territories increased when PF synapses were structurally reduced in mice lacking GluD2 or Cbln1 (Ichikawa et al., 2002; Hirai et al., 2005). Furthermore, CF territories were reduced by inhibiting PF synaptic transmission in the deep ML, but not in the superficial ML (Park et al., 2019). Taken together, it is possible that the physical competition of CF synapses with PF synapses limits CF territories within appropriate regions, while functional PF inputs at the deeper ML promote CF territory elongation.

Another major input, MFs, originates from multiple nuclei in the brainstem and spinal cord. Their arrivals in the developing cerebellum vary according to their origins, yet all types of MFs arrive by P0 (Rahimi-Balaei et al., 2015). Although the EGL includes precursors of GCs, which MFs innervate in the mature cerebellum, MFs do not enter the EGL and wait for GCs to differentiate and migrate into the IGL. Meanwhile, MF terminals increase in size to form characteristic mature MF terminals (Hámori and Somogyi, 1983; Kim et al., 2023), called rosettes. On the other hand, some MFs originating from the pontine nucleus or spinal cord transiently contact PCs during this developmental period (Kuwako et al., 2014; Sillitoe, 2016). Around the third postnatal week, MF synaptic connections are finally established with the dendrites of GCs and Golgi cells in the glomeruli in the IGL.

The MFs originating from the spinal cord, labeled by an anterograde tracer dye, were present in the agranular cerebellum (van der Heijden et al., 2021), suggesting that GCs are not required for the arrival of MFs in the cerebellum. In contrast, GCs are easily expected to have a significant impact on MF development inside the cerebellum, because synaptogenesis often relies on the interplay between pre- and post-synaptic neurons (Akins and Biederer, 2006; Petzoldt and Sigrist, 2014). Indeed, studies have revealed the role of GCs and their molecules in MF development (Figure 3E). An early electron microscopy study first suggested the role of GCs by demonstrating the correlation between rapid MF enlargement and intense increase in GC dendrites (Hámori and Somogyi, 1983). The increase in size and presynaptic differentiation of MF terminals, the latter of which can be observed by clustering of synaptic vesicles or presynaptic molecules, have been shown to be mediated by secreted molecules from GCs, WNT-7a, and fibroblast growth factor 22 (FGF22), and by postsynaptically localized membrane protein in GCs, neuroligins (Hall et al., 2000; Scheiffele et al., 2000; Umemori et al., 2004). Furthermore, another study demonstrated the role of a cell adhesion molecule, cadherin-7 (Cdh7), expressed in MFs and GC dendrites: Cdh7 regulates MF axonal growth termination in the IGL and specific synapse formation between MFs and dendrites of GCs through homophilic binding (Kuwako et al., 2014). These molecules released from GCs or expressed in GC membranes are likely critical for synaptic formation and/or maturation not just between GCs and MF terminals originally located in the IGL but also between GCs and MF terminals that transiently make contact with PCs and are subsequently eliminated.

One of the characteristic anatomical features of GCs is that their PFs are stacked in the ML, and as a result, each of them is located in a specific sublayer of the ML. Early studies using Golgi staining suggested correlations between the locations of GC somas in the IGL and the locations of their PFs in the ML, as well as the projections of MFs originating from specific nuclei to specific sublayers of the IGL (Eccles et al., 1967; Altman, 1982), leading to the hypothesis that signals arising from MFs of different origins would be conveyed to different sublayers of the ML through activated PFs. However, later studies demonstrated no apparent correlations between GC soma locations and their PF locations (Zong et al., 2005; Wilms and Häusser, 2015; Markwalter et al., 2019; Rhee et al., 2021), which required a revisiting of this hypothesis. Recent studies using advanced labeling techniques have finally demonstrated the presence of structured synaptic connections between the specific origin of MFs and dendrites of GCs that have PFs at specific sublayers of the ML (Shuster et al., 2021; Kim et al., 2023). Furthermore, it has been suggested that such arrangement results from the synaptic formation between partners of MFs and GC dendrites that have matched developmental timing (Kim et al., 2023). Thus, the expression of the abovementioned molecules involved in synapse formation and maturation may be temporally controlled during postnatal development, contributing to the formation of a structured network.

### 3.4. GC–GC interaction

As described earlier in this article, GCs go through dynamic developmental processes, namely the proliferation of GCPs in the RL and the EGL, tangential migration in the EGL, radial migration through the ML and the PCL, and maturation in the IGL. The sequential expression of many molecules in developing GCs has been shown to be crucial for the development of GCs themselves (Vaudry et al., 2003; Leto et al., 2016; Lackey et al., 2018; Iulianella et al., 2019; Wang and Liu, 2019; Consalez et al., 2021). Since this article focuses on the GC-dependent regulation of cerebellar network formation, we discuss interactions of GCs that affect the development of other GCs. One likely interaction would occur during GC radial migration in the ML (Figure 2E). The N-methyl-D-aspartate (NMDA) type of glutamate receptor was shown to accelerate GC migration through nonsynaptic activation (Komuro and Rakic, 1993). Although the sources of glutamate are not clarified, PFs may be one of them, considering that GCs migrate through previously developed PFs in the ML. GC migration is also promoted by BDNF, as mice lacking BDNF showed impaired migration (Borghesani et al., 2002). During postnatal development, *Bdnf* mRNA is expressed in GCs of the IGL (Rocamora et al., 1993). In addition, a study revealed the generation of a BDNF gradient, with increasing BDNF levels along the migration path from the EGL to the IGL, which plays a crucial role in guiding GC migration (Zhou et al., 2007). This suggests that BDNF released by previously developed GCs promotes the migration of other GCs. BDNF also appears to stimulate autocrine release of BDNF from migrating GCs, thereby further amplifying the BDNF gradient and enhancing migration (Zhou et al., 2007).

After an extensive series of cell divisions of GCPs in the outer EGL, GCPs in the inner EGL exit mitosis and commence their migration. The cell cycle exit of GCPs appears to be regulated by several kinds of molecules, such as transcriptional regulators, membrane proteins, growth factors, or signaling molecules (Penas et al., 2019; Kullmann et al., 2020; Adachi et al., 2021; Consalez et al., 2021; Miyashita et al., 2021; Van Battum et al., 2021; Zanin and Friedman, 2022; Watanabe et al., 2023). When GCPs exit the cell cycle, these molecules are presumably upregulated, while molecules involved in proliferation are downregulated. An important question arises regarding the initiation of such upregulation and downregulation. It was reported that increasing oxygen tension is a critical switch for cell cycle exit (Kullmann et al., 2020). During the early postnatal days, limited vascularization led to the expression of hypoxia-inducible factor 1α (Hif1α) in GCPs, subsequently inhibiting the differentiation of GCs. As vascularization advanced later on, these inhibitory mechanisms were downregulated. While this study offers valuable insight, the timing of vascularization alone may not fully explain the gradual GC differentiation during the initial two postnatal weeks. A study showed that GCPs proliferate in the microenvironment of the outer EGL, and migration away from such a mitogenic niche of the outer EGL promotes cell cycle exit (Choi et al., 2005), providing the possibility that signals in the outer EGL trigger the migration of GCPs toward the inner EGL and, in turn, facilitate cell cycle exit. These signals may function by reducing the expression of molecules that prevent GCP migration, such as the p75 neurotrophin receptor (Zanin and Friedman, 2022). Given the high density of proliferating GCPs in the outer EGL, such signals may be derived from the interactions between GCPs. A recent study using electron microscopy analysis revealed a unique way of interactions between developing GCs in the EGL through intercellularly connecting structures (Cordero Cervantes et al., 2023), although the functions of these structures remain to be elucidated.

## 4. Conclusion and perspectives

It is progressively being recognized that GCs play an important role not only as essential components for information processing in the mature cerebellum but also as critical regulators of cerebellar network construction during development. We discussed such GC-dependent regulation of cerebellar development, as summarized in Table 1. As seen in this article, the majority of the regulation is also related to, mediated by, or occurring concurrently with GCs’ own development. The developmental processes of GCs are also supported by other developing components in the cerebellum, and consequently, they can reciprocally regulate each other. Thus, GCs are not only crucial regulators of cerebellar network formation. Nevertheless, it would be particularly interesting to understand how GCs contribute to the cerebellar network formation, considering three properties of GCs. First, GCs, including GCPs and PFs, occupy large areas of the cerebellar cortex throughout cerebellar development. Secondly, a substantial number of developing GCs travel dynamically from the surface to the inside of lobules. Third, GCs are the only major excitatory neurons in the cerebellar cortex. We would like to close this article by proposing four prospective research directions that could help us better understand the GC-dependent regulation of cerebellar development.

1. In our exploration of the GC-dependent regulation of cerebellar development, we have also raised remaining questions in this article. The questions include understanding the determinants of locally differential control of GCP proliferation, which in turn impact the cerebellar gross structures; elucidating the role of microdomain coupling in PF boutons for PC development; examining potential cues for MLI differentiation in the pWM; unraveling the temporal control of molecule expression that regulates MF-GC synaptic connections; and uncovering signals that dictate the cell cycle exit of GCPs. Addressing these questions would stand as a primary direction.
2. Granule cells may separately regulate the development of individual components, such as specific types of neurons or synapses, as we mostly discussed in this article. However, considering the abovementioned three properties of GCs, GCs might be appropriate regulators that systematically orchestrate the overall cerebellar network formation. Particularly, it is interesting to test whether there is coordinated network formation between the ML and IGL, and if so, whether GCs are involved in its regulation. An example of such coordination would be the structured synaptic connections between MFs originating from specific origins and dendrites of GCs having PFs in specific areas of the ML (Shuster et al., 2021; Kim et al., 2023).
3. In this article, we discussed several elements of cerebellar development, such as the development of PCs and MLIs, or the establishment of CF and MF inputs. However, there are other types of neurons and glial cells besides GCs, PCs, and MLIs (Schilling et al., 2008; Hull and Regehr, 2022), and neuromodulatory projections are also present in the cerebellum (Jaarsma et al., 1997; Li et al., 2014; Oostland and van Hooft, 2016; Zitnik et al., 2016). In addition, synaptic connections within the cerebellar cortex are more complex than previously understood (Hull and Regehr, 2022). While our understanding of the developmental regulation of other types of neurons, projections, and synaptic connections is limited at the moment, it is possible that GCs and their molecules coordinate the incorporation of these elements into cerebellar networks, taking into account the three properties of GCs stated above.
4. The involvement of GCs in certain aspects of cerebellar development could be elucidated using molecule deficits, as has been done frequently, and transcriptional analyses would provide valuable insights into identifying prospective molecules. In general, spatiotemporal patterns of gene expression are precisely regulated during development, and such a coordinated gene expression program is critical for appropriate brain development. In other words, molecules exhibiting dynamic changes in expression during certain times are expected to function in developmental events occurring at that time. Indeed, it has been shown that the expression of many molecules required for specific GC developmental events is tightly regulated in GCs at specific developmental stages (Consalez et al., 2021). Thus, spatiotemporal expression patterns of molecules in developing GCs would provide predictions regarding the involvement of the molecules not only in GC developmental events but also in the GC-dependent regulation of cerebellar network formation. A study predicted the temporal patterns of gene expression during postnatal GC development by creating pseudotime ordering of developing GCs based on single-nucleus RNA sequencing (snRNA-seq) data (Rosenberg et al., 2018). Among the molecules with differential expression across the pseudotime, those increasing at late stages are likely critical for mature GC functions, but some of them may also contribute to cerebellar network formation, which is actively ongoing in the late postnatal developmental period. Particularly, secreted molecules and membrane molecules are interesting to be tested, considering their potential abilities for cell–cell interactions. Although the temporal patterns of individual gene expression were not analyzed, snRNA-seq was also utilized for the characterization of cell types in the human fetal cerebellum (Aldinger et al., 2021). The study demonstrated the enrichment of neurodevelopmental-disorder risk genes in multiple cell types, including GCPs and GCs, raising the possibility that molecules encoded by the GC-enriched genes may contribute to cerebellar functional development. In addition, gene expression in developmentally synchronized GCs was analyzed by *in vivo* electroporation and translating ribosomal affinity purification (Yang et al., 2016). It was demonstrated that the NuRD chromatin-remodeling complex inactivates activity-dependent genes, such as c-fos or nr4a1, around the time window of GC dendrite morphogenesis, and the inactivation of these genes indeed regulates GC dendrite pruning. Since mature GCs have only 3–5 dendrites, and thus dendrite pruning is a critical step for GC maturation, the regulation of activity-dependent genes and their downstream molecules presumably also affects synaptic formation or cerebellar network formation. Utilizing data derived from different types of gene expression analyses may lead to a deeper understanding of how GCs and their molecules regulate the development of cerebellar networks.

## Author contributions

MK and SJ wrote the manuscript draft and made the figures. HP edited the manuscript and figures. YY and KT-Y conceptualized, wrote the manuscript draft, and edited the manuscript. All authors contributed to the article and approved the submitted version.

## Funding

This work was supported by the KIST Institutional Program (project no.: 2E32211) and the National Research Foundation of Korea (NRF) grant funded by the Korean Ministry of Science and ICT (NRF grant nos.: 2021R1A2C3009991, 2021R1C1C2007843, and 2022R1A2C2006857).
